# An Allometric Approach to Fine-Mapping Subcortical Volume Alterations in RASopathies

**DOI:** 10.1523/ENEURO.0017-26.2026

**Published:** 2026-09-18

**Authors:** Chloe A. McGhee-Deakin, Odeya Russo, Julia R. Plank, Tamar Green

**Affiliations:** ^1^Department of Psychiatry & Behavioral Sciences, Stanford University, Stanford 94305; ^2^Institute for Behavioral Genetics, University of Colorado, Boulder, Colorado 80309; ^3^Department of Psychology and Neuroscience, University of Colorado, Boulder, Colorado 80309; ^4^Department of Pediatrics, Stanford University, Stanford 94305

**Keywords:** allometry, morphometry, neurofibromatosis type 1, Noonan syndrome, Noonan syndrome with multiple lentigines, RASopathies

## Abstract

Structural neuroimaging in youth with RASopathies has shown alterations in total brain volume (TBV) and subcortical structures. However, whether these subcortical differences scale proportionately with TBV or follow nonlinear allometric relationships remains unclear, limiting accurate interpretation of neuroanatomical findings and their relevance to cognition. Using an allometric framework, this study aimed to determine whether (1) subcortical volumes exhibit deviations from normative scaling in RASopathies and (2) methods used to account for TBV influence the detection of subcortical volume alterations. We examined youth with RASopathies (*N* = 132), including neurofibromatosis type 1 (NF1; *N* = 27), Noonan syndrome (*N* = 100; *PTPN11*, 72; *SOS1*, 22; *RAF1*, 6), and Noonan syndrome with multiple lentigines (NSML; *N* = 5), compared with 80 age- and sex-matched controls. Subcortical volumes were analyzed using an allometric framework, compared with linear TBV correction methods (normalization and covariation), and explored in sex-stratified analyses. Allometric analyses revealed deviations from normative scaling in the thalamus for NF1; the caudate, putamen, ventral striatum, and pallidum for *PTPN11*; and the ventral striatum for *SOS1*. No significant deviations were observed for NSML or *RAF1*. Exploratory sex-stratified analyses suggested similar directional scaling patterns across sexes, with greater deviations in females, although subgroup sizes were small. Comparisons across correction methods demonstrated that linear approaches may overestimate regional differences, whereas thalamic alterations in NF1 and striatal alterations in *PTPN11* were consistent across methods, suggesting robust, region-specific genetic effects beyond scaling. These findings highlight genotype-specific deviations from normative allometric scaling and underscore the importance of nonlinear scaling frameworks to accurately characterize neuroanatomical differences in RASopathies.

## Graphical abstract

**Figure:**
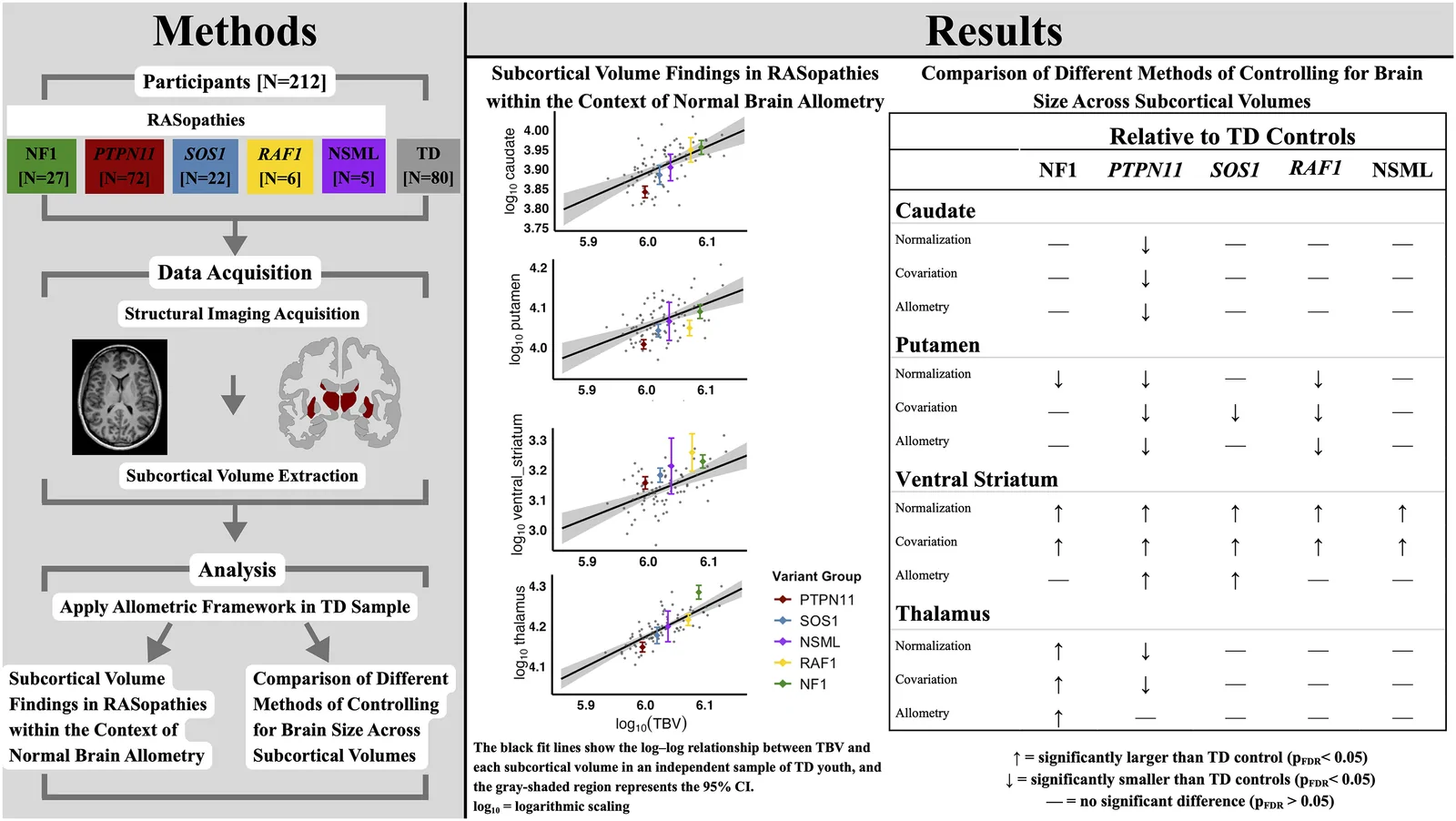
Visual Abstract

## Significance Statement

Neurofibromatosis type 1 and Noonan Syndrome Spectrum Disorders are associated with atypical brain size, yet most neuroimaging studies rely on linear correction methods that assume proportional brain scaling. Regional brain allometry poses an important but underappreciated methodological challenge for accurately characterizing neuroanatomical differences in these conditions. Here, we apply a normative allometric framework that reveals gene-specific and preliminary sex-specific deviations in subcortical scaling that are potentially overestimated by linear correction approaches. These findings refine the interpretation of brain structure differences in RASopathies and demonstrate the need to account for nonlinear scaling in neurodevelopmental research. Incorporating allometric approaches will improve the biological specificity of neuroimaging markers and inform future efforts toward personalized assessment and intervention.

## Introduction

RASopathies are genetic disorders caused by germline variants in the RAS/MAPK pathway (Tartaglia and Gelb, 2010; Tidyman and Rauen, 2016a). This group includes neurofibromatosis type 1 (NF1; prevalence 1:2,600–3,000), caused by loss-of-function variants in NF1 (Jafry and Sidbury, 2020; Anastasaki et al., 2022), and Noonan syndrome (NS; prevalence ∼1:2,000), the most common RASopathy. NS is primarily due to missense variants in *PTPN11* (∼50%), *SOS1* (∼10%), and *LZTR1* (∼10%), with other genes accounting for an additional 10–20%, all enhancing RAS/MAPK signaling (Tartaglia et al., 2011, 2022; Roberts et al., 2013). Noonan syndrome with multiple lentigines (NSML), allelic to NS, arises from distinct *PTPN11* missense variants that impair SHP2 activity and dysregulate signaling, including PI3K-AKT-mTOR (Digilio et al., 2002; Bonetti et al., 2014; Hodgson et al., 2024). Despite genetic and clinical heterogeneity, RASopathies disrupt neurodevelopment, making them a valuable model for studying genetic influences on brain structure.

Clinically, RASopathies are associated with a range of neurodevelopmental and cognitive difficulties, including attention problems, executive dysfunction, learning disabilities, motor impairments, and autistic traits (Pierpont et al., 2013, 2015; Pierpont, 2016; Johnson et al., 2019; McNeill et al., 2019; Foy et al., 2022; Naylor et al., 2023; McGhee et al., 2025; Serur et al., 2025b). Because subcortical structures such as the striatum, thalamus, hippocampus, and amygdala play central roles in executive functioning, motor control, memory, and socioemotional processing (Lanciego et al., 2012; Koziol et al., 2014; Palomero-Gallagher and Amunts, 2022), characterizing subcortical volume alterations may provide insight into the neural mechanisms underlying these phenotypes. Supporting this framework, neuroimaging studies of neurodevelopmental disorders, including autism spectrum disorder and attention-deficit/hyperactivity disorder, have identified alterations in subcortical structures, including the striatum, nucleus accumbens, amygdala, and hippocampus, that are associated with cognitive, behavioral, and emotional functioning (Hoogman et al., 2017; van Rooij et al., 2018; Faraone et al., 2021), suggesting that subcortical morphology may represent an important neural correlate of neurodevelopmental and cognitive outcomes. In RASopathies, previous research has identified similar subcortical volume alterations relative to typically developing (TD) individuals. In NS, reductions appear in the bilateral striatum (caudate, putamen, and pallidum), right hippocampus, and right thalamus (Johnson et al., 2019; Rai et al., 2023; McGhee et al., 2024). Variant-specific effects show *PTPN11* variants with pronounced reductions in the striatum, hippocampus, right thalamus, and amygdala, while *SOS1* variants show subtler reductions with enlarged ventral striatum (Johnson et al., 2019; Rai et al., 2023; Serur et al., 2025a). NSML shows similar patterns (Serur et al., 2025a), whereas NF1 exhibits larger hippocampus, amygdala, and thalamus volumes (Violante et al., 2013; Barkovich et al., 2018; Wang et al., 2022; McGhee et al., 2024). Importantly, these studies compared RASopathies only with TD groups, without directly comparing multiple RASopathy-associated variants.

Despite robust reports of subcortical volumetric alterations in RASopathies (Violante et al., 2013; Barkovich et al., 2018; Johnson et al., 2019; Wang et al., 2022; Rai et al., 2023; McGhee et al., 2024; Serur et al., 2025a), it remains unclear whether these differences reflect region-specific effects of pathogenic RAS/MAPK genetic variants or are also driven by the scaling method used to account for total brain volume (TBV). This distinction is particularly important because RASopathies are associated with systematic differences in overall brain size, with NF1 typically exhibiting increased TBV and NS exhibiting reduced TBV relative to TD individuals (Reardon et al., 2016; Nadig et al., 2018; Janssen et al., 2022). Consequently, apparent subcortical alterations may arise either from genetic effects on regional neurodevelopment or from the way subcortical structures scale with overall brain size.

Most prior studies have addressed TBV differences using normalization or covariation approaches that assume a linear relationship between regional volumes and TBV (Violante et al., 2013; Barkovich et al., 2018; Johnson et al., 2019; Rai et al., 2023; McGhee et al., 2024). However, growing evidence indicates that brain structures scale nonlinearly with brain size and that neurogenetic conditions characterized by altered TBV often exhibit distinct allometric patterns (Reardon et al., 2016; Nadig et al., 2018; Janssen et al., 2022). In such contexts, linear correction methods may overestimate or obscure region-specific effects.

Allometric analyses provide a framework for addressing this issue by modeling how brain structures scale relative to the overall brain size (Reardon et al., 2016; Nadig et al., 2018; Janssen et al., 2022). This approach has revealed clinically meaningful patterns in other conditions characterized by atypical brain size. For example, in sex chromosome aneuploidies, allometric analyses identified negative deviations from normative scaling in the pallidum, striatum, amygdala, hippocampus, and thalamus, indicating disproportionately smaller-than-expected volumes relative to TBV (Reardon et al., 2016; Nadig et al., 2018). Similarly, although individuals with schizophrenia exhibit subcortical volumes that generally conform to normative scaling relationships at baseline, longitudinal analyses have revealed disproportionately greater age-related sulcal declines (Janssen et al., 2022). Together, these studies highlight the importance of allometric approaches for conditions with atypical brain size, capturing nonlinear scaling and region-specific effects that linear correction methods may overestimate.

Given that RASopathies are associated with substantial alterations in TBV (Violante et al., 2013; Johnson et al., 2019; Wang et al., 2022), they may similarly exhibit nonlinear scaling relationships that are not adequately captured by linear correction approaches. An allometric framework, therefore, offers an opportunity to better capture the nonlinear biological processes underlying brain growth and organization, providing a more accurate characterization of how genetic variations in RASopathies affect subcortical structures relative to overall brain size.

In the present study, we used an allometric framework to determine whether (1) subcortical volumes show allometric deviations from normative scaling, which provides insights into the biological scaling of subcortical structures in RASopathies, and (2) the methods used to account for TBV influence the detection of subcortical volume alterations in RASopathies. We examined youth with RASopathies (*N* = 132), including NF1 (*N* = 27), NS (*N* = 100; *PTPN11*, 72; *SOS1*, 22; *RAF1*, 6), and NSML (*N* = 5), alongside age- and sex-matched TD youth (*N* = 80). Using the TD cohort, we first generated normative models describing the scaling of subcortical structures relative to TBV. We then tested whether RASopathy groups deviated from these normative scaling relationships compared with TD individuals, as such deviations would indicate that subcortical volumes differ from those expected based on normative brain–structure scaling patterns. Although there is currently no established evidence of clinically meaningful sex differences in RASopathies, evidence from TD populations indicates sex differences in subcortical brain structure (Sowell et al., 2002; Giedd et al., 2012; Duerden et al., 2020). Given our relatively large RASopathy neuroimaging cohort, we additionally conducted exploratory sex-stratified analyses to examine whether similar patterns might be present in RASopathies. Finally, we compared allometric findings with linear correction methods to evaluate whether previously reported subcortical volume differences represent robust genetic effects or artifacts of linear scaling assumptions.

## Materials and Methods

### Participants

All participants were recruited over seven years (2018–2025) across the United States and Canada and brought to Stanford for a single-site research study. Youth with RASopathies (i.e., NF1, NS, and NSML) were recruited across the United States and Canada through the Noonan Syndrome Foundation and Stanford University School of Medicine's website, parent social media networks, and advertisements, while TD youth were recruited locally in the California Bay Area.

Youth with NF1, NS, NSML, and TD were excluded if they met any of the following criteria: known diagnosis of a major psychiatric disorder (e.g., severe psychotic disorders, such as psychosis or major mood disorders, or previously documented full-scale IQ < 70), a current neurological disorder (e.g., seizures, history of head trauma, a diagnosis of gross structural brain malformations, or any known tumors or cerebellar, brainstem, tectal plate, and basal ganglia gliomas), any MRI scan contraindications (the presence of nonremovable metal including braces, severe claustrophobia), premature birth (gestational age ≤34 weeks), or birthweight <2,000 g. TD participants were selected to group-match the RASopathy genetic variant groups in terms of age and sex. The Institutional Review Board of the Stanford University School of Medicine approved all procedures in this study. Written consent was obtained from the legal guardians of all participants, and participants over the age of seven provided written assent. 

Eligible participants included children aged 4 to 17 years diagnosed with NF1 (*N* = 27; mean age, 9.12; females, 13), Noonan Syndrome Spectrum Disorder (NSSD) including youth diagnosed with either NS or NSML (*N* = 105; mean age, 10.00; females, 57) and TD youth (*N* = 80; mean age, 9.54; females, 46). All participants in the NF1 and NSSD groups provided a genetic test report identifying a pathogenic or likely pathogenic variant consistent with their diagnosis. All genetic reports were reviewed by a study clinician/geneticist to confirm eligibility. Children in the NSSD group were diagnosed based on variants in the following genes: *PTPN11* (*N* = 72 for NS; *N* = 5 for NSML), *SOS1* (*N* = 22 for NS), and *RAF1* (*N* = 6 for NS). Specifically, for *PTPN11*, 11 cases were inherited, 15 were de novo, and 46 had an unknown status. For NSML, two cases were de novo, and three had an unknown status. In *SOS1*, four cases were inherited, six were de novo, and 12 had an unknown status. In *RAF1*, there were two inherited cases, one case was de novo, and three cases had an unknown status. In the NF1 gene, there were 22 de novo cases and five cases with unknown status.

The neurodevelopmental, cognitive, and behavioral profiles of a subset of youth in the current group have been previously reported (Johnson et al., 2019; Naylor et al., 2023; McGhee et al., 2024; Plank et al., 2024; Siqueiros-Sanchez et al., 2024; Serur et al., 2025a).

### Structural data imaging acquisition and preprocessing

We collected structural MR imaging data of subjects in all groups (NF1, NS, NSML, and TD) at the Lucas Center at Stanford University using a GE 3 T whole-body MR system (GE Healthcare). All data were collected using one of two compatible T1-weighted imaging protocols. In the first protocol (participants enrolled between 2018 and 2022), 76 subjects were scanned using a GE Discovery 750 scanner equipped with an eight-channel head coil. The pulse sequence employed was fast spoiled gradient-recalled echo, with slices acquired in the sagittal direction, and the following parameters: echo time (TE), 3.18 ms; inversion time (TI), 459 ms; repetition time (TR), 816 ms; voxel size, 1.0 ´ 1.0 ´ 1.0 mm; flip angle, 12°; matrix, 256 ´ 256; 176 slices; field of view (FOV), 256 ´ 256 mm; and acquisition time (TA), 4 min 30 s. These data were processed using FreeSurfer (https://surfer.nmr.mgh.harvard.edu version 5.3).

In the second protocol (participants enrolled between 2022 and 2024), a total of 136 participants were scanned using a GE Signa Premier scanner equipped with a 48-channel head coil. The pulse sequence employed was a magnetization-prepared rapid gradient-echo, with slices acquired in the axial direction, and the following parameters: TE, 2.83 ms; TI, 900 ms; TR, 1,993 ms; voxel size, 1.0 ´ 1.0 ´ 1.2 mm; flip angle, 8°; matrix, 240 ´ 240; 146 slices; FOV, 240 ´ 240 mm; and TA, 4 min 24 s.

These data were processed using FreeSurfer (version 7.2.0). The pipeline included removal of the nonbrain tissue using a hybrid surface deformation procedure, segmentation of the subcortical white matter and deep gray matter volumetric structures, intensity normalization, tessellation of the gray matter–white matter boundary, and automated topology correction (Oliveira et al., 2010). Cortical maps of the brain surfaces for each hemisphere create 34 region parcellations per hemisphere (Desikan et al., 2006). 

All FreeSurfer surfaces were visually inspected, edited, and finalized according to strict lab protocols by trained editors (M.R. and C.A.M-D.). In particular, gray–white matter boundaries were carefully examined and manually adjusted when necessary. Image quality was considered unusable if artifacts were present due to subject motion, blood flow, or wraparound. Based on these criteria, two participants with NS and two with NF1 were excluded from the analyses.

To mitigate systematic differences associated with the scanner, head coil, pulse sequence, and FreeSurfer version, we applied ComBat harmonization to regional subcortical volumes and TBV. ComBat is a Bayesian framework that adjusts for batch effects in multisite or multiprotocol imaging data while preserving biological variability (Fortin et al., 2017, 2018; Radua et al., 2020). Batch was defined as the imaging acquisition and processing protocol, including scanner differences and FreeSurfer versions (first vs second processing pipeline). Region-of-interest (ROI) volumes were harmonized using ComBat prior to statistical analyses while preserving variance associated with age, sex, and diagnostic group. To evaluate the robustness of the main findings, we re-estimated the normative allometric models at the TD level, including the imaging acquisition/processing pipeline as a covariate (scanner and FreeSurfer version; Extended Data Table 3-2).

For all analyses, subcortical regions were defined using the Desikan–Killiany parcellation (Desikan et al., 2006), including bilateral caudate, putamen, pallidum, thalamus, hippocampus, amygdala, and nucleus accumbens (also referred to as the ventral striatum).

### Statistical analysis

All statistical analyses were performed in R v4.4.1 (R Core Team, 2024).

#### Descriptive analysis

We conducted *t* tests or Mann–Whitney *U* tests to examine differences in age and TBV among the NSSD, NF1, and TD groups. The choice of statistical test was based on the normality of the data, as determined by Shapiro–Wilk tests. The *χ*^2^ tests or Fisher's exact tests were used to determine differences in sex, ethnicity, and race distributions across the groups. Counts and percentages for race and ethnicity were reported for each subgroup.

#### TBV differences across RASopathies

To explore the influence of RASopathy-associated genetic variants (NF1, *PTPN11*, *SOS1*, *RAF1*, and NSML) on TBV, we first conducted an omnibus analysis of variance. Age and sex were included as covariates. Prior to interpreting the results, we confirmed that model assumptions were met, including the independence of observations, normality of residuals, and homogeneity of variance across groups. When the omnibus test indicated a significant overall effect, we performed post hoc pairwise comparisons on the estimated marginal means of TBV between genetic variant groups. To account for multiple comparisons, we applied a false discovery rate (FDR) correction (Benjamini and Hochberg, 1995).

#### Allometry of subcortical volumes

To investigate the relationships between TBV and each subcortical volume, we utilized the previously established allometric framework (Reardon et al., 2016; Nadig et al., 2018). We utilized our sample of 80 TD youth to establish normative scaling relationships for each subcortical volume using the following equation. These normative allometric relationships were estimated at the group level using the TD sample, providing a single scaling coefficient (*β*_1_) for each subcortical structure rather than individual-level scaling estimates:

$$\mathrm{lo}g_{10}(\mathrm{Subcortical}\;\mathrm{Volume})\;=\;\beta_{0}\;+\;\beta_{1}\times \mathrm{lo}g_{10}(\mathrm{TBV})\;+\varepsilon .$$

This log–log regression equation was used to allow for a nonlinear representation of the relationship between subcortical volume and TBV. In this equation, the scaling coefficient *β*_1_ quantifies the relationship between variations in TBV and the corresponding variations in subcortical volumes. A linear scaling relationship is indicated by a *β*_1_ value of 1, which represents isometric scaling, meaning that the proportional volume of the subcortical structure remains constant regardless of variations in TBV. In contrast, hypoallometric scaling is represented by *β*_1_ values <1, indicating disproportionately smaller subcortical volumes relative to TBV at higher TBV values. Lastly, hyperallometric scaling, reflected by *β*_1_ values >1, indicates disproportionately larger subcortical volumes relative to TBV at higher TBV values. To determine if computed *β*_1_ values significantly deviated from isometric scaling, we utilized a one-sample *t* test and reported the FDR-corrected *p* values. As sensitivity analyses, the normative allometric models in the TD sample were re-estimated with additional covariates. First, age was included as a covariate to assess whether the observed scaling relationships were robust to age-related variation (Extended Data Table 3-1). Second, the normative allometric models were re-estimated with the imaging acquisition/processing pipeline included as a covariate (scanner and FreeSurfer version) to evaluate whether the observed scaling relationships were robust to variation in image acquisition and processing (Extended Data Table 3-2).

Utilizing the established group-level normative scaling laws for each subcortical structure, we evaluated whether the observed subcortical volumes in each genetic variant group (i.e., *PTPN11*-NS, *SOS1*, NSML, *RAF1*, and NF1) either fell within the predicted allometric ranges established by the TD group or represented statistically significant deviations from these normative relationships. A significant deviation was defined as the absence of overlap between the 95% confidence intervals of the observed subcortical volumes in the genetic variant groups and the predicted allometric ranges from the TD group (Reardon et al., 2016; Nadig et al., 2018).

#### Sex-stratified allometric analyses of subcortical volumes

We conducted exploratory sex-stratified analyses within the allometric framework to assess whether deviations from normative subcortical scaling differed between males and females. Using TD participants, we estimated sex-specific normative scaling relationships between subcortical volumes and TBV separately in males and females, applying the same analytical steps described above. These models were then used to derive expected subcortical volumes for each participant.

In RASopathy groups, deviations from normative scaling were quantified as the difference between observed and TD-predicted values, divided by the residual standard deviation of the corresponding sex-specific TD model, with positive values indicating larger-than-expected volumes and negative values indicating smaller-than-expected volumes relative to normative scaling expectations. These *z*-scores provide a standardized measure of the magnitude and direction of deviation from normative allometric expectations, facilitating interpretation of their potential clinical relevance.

All sex-stratified analyses were exploratory and hypothesis-generating. Given sample size considerations, these analyses were restricted to *PTPN11* (*N* = 72), *SOS1* (*N* = 22), and NF1 (*N* = 27).

#### Linear and allometric scaling frameworks for subcortical volume analysis

To determine whether the method used to account for TBV influences the detection of subcortical volume alterations in RASopathies*,* we compared results from the allometric method with normalization and covariation methods, both of which assume linear scaling between subcortical volumes and TBV. These analyses compared subcortical volumes in each RASopathy-associated genetic variant group with those in TD youth.

The normalization method utilized a linear regression model with normalized subcortical volume (defined as subcortical volume divided by TBV) as the outcome and genetic variant group as the predictor:

Normalization:
$$(\mathrm{Subcortical}\;\mathrm{Volume}\;/\;\mathrm{TBV})\;=\;\beta_{0}\;+\;\beta_{1}\times (\mathrm{Genetic}\;\mathrm{Variant}\;\mathrm{Group})\;+\varepsilon .$$

The covariation method also employed a linear regression model, with subcortical volume as the outcome, genetic variant group as the predictor, and TBV as a covariate:

Covariation:
$$\mathrm{Subcortical}\;\mathrm{Volume}\;=\;\beta_{0}\;+\;\beta_{1}\times (\mathrm{Genetic}\;\mathrm{Variant}\;\mathrm{Group})\;+\;\beta_{2}\times (\mathrm{TBV})\;+\varepsilon .$$

In both the normalization and covariation methods, the genetic variant group was treated as a binary predictor, comprising the genetic variant of interest and the TD control group. Because each subcortical region was tested across five genetic variant groups for each method, we applied FDR correction to adjust for multiple comparisons.

Finally, we used the normalization and covariation methods to compare subcortical volumes directly across RASopathy genetic variant groups, rather than relative to TD controls, to evaluate whether patterns of subcortical differences were consistent across genetic variant groups. If a significant difference was detected, we performed post hoc pairwise *t* tests to identify specific differences between the genetic variant groups, applying an FDR correction for multiple comparisons.

## Results

### Demographic characteristics

The NSSD, NF1, and TD groups did not differ in age or sex (Table 1). As expected, the three groups exhibited significantly different TBV, with results indicating that compared with TD, the NSSD group had reduced TBV (*p* = 0.021), and the NF1 group had enlarged TBV (*p* < 0.001) after controlling for age and sex (Table 1). Furthermore, no significant differences in ethnicity or race were identified between the NF1 and TD groups or between the NSSD and NF1 groups. In contrast, the NSSD and TD groups showed significant differences in both race and ethnicity, with the TD group having a higher representation of Hispanic/Latino and Asian youth, while the majority of the NSSD group was White and NonHispanic.

**Table 1.** Participant demographic information

	*PTPN11* (*N* = 72)	*SOS1* (*N* = 22)	*RAF1* (*N* = 6)	NSML (*N* = 5)	NSSD (*N* = 105)	NF1 (*N* = 27)	TD (*N* = 80)	Overall (*N* = 212)	NSSD versus TD	NF1 versus TD	NSSD versus NF1
Sex											
Female	39 (54.2%)	13 (59.1%)	1 (16.7%)	4 (80.0%)	57 (54.3%)	13 (48.1%)	46 (57.5%)	116 (54.7%)	0.774^a^	0.535^a^	0.724^a^
Male	33 (45.8%)	9 (40.9%)	5 (83.3%)	1 (20.0%)	48 (45.7%)	14 (51.9%)	34 (42.5%)	96 (45.3%)			
Age											
Mean (SD)	10.1 (3.07)	9.19 (3.19)	12.6 (3.42)	9.45 (2.81)	10.0 (3.15)	9.12 (2.23)	9.54 (2.41)	9.74 (2.79)	0.360^b^	0.360^b^	0.221^b^
Median [Min, Max]	10.2 [4.43, 17.7]	8.01 [5.71, 16.3]	12.0 [9.04, 17.5]	10.1 [6.05, 13.4]	9.85 [4.43, 17.7]	8.31 [6.11, 13.8]	9.43 [4.05, 16.8]	9.48 [4.05, 17.7]			
Ethnicity											
Hispanic/Latino	6 (8.3%)	0 (0.0%)	0 (0.0%)	0 (0.0%)	6 (5.7%)	4 (14.8%)	13 (16.3%)	23 (10.8%)	0.026*^c^	1.00^c^	0.091^c^
Not Hispanic/Latino	65 (90.3%)	22 (100%)	6 (100%)	5 (100%)	98 (93.3%)	20 (74.1%)	65 (81.3%)	183 (86.3%)			
Unreported	1 (1.4%)	0 (0.0%)	0 (0.0%)	0 (0.0%)	1 (1.0%)	3 (11.1%)	2 (2.5%)	6 (2.8%)			
Race											
Asian	3 (4.3%)	0 (0.0%)	0 (0.0%)	0 (0.0%)	3 (2.9%)	2 (7.4%)	11 (13.8%)	16 (7.5%)	0.004*^c^	0.379^c^	0.135^c^
Black/African American	2 (2.8%)	0 (0.0%)	0 (0.0%)	0 (0.0%)	2 (1.9%)	1 (3.7%)	0 (0.0%)	3 (1.4%)			
Multiracial	7 (9.7%)	2 (9.1%)	0 (0.0%)	0 (0.0%)	9 (8.6%)	4 (14.8%)	12 (15.0%)	25 (11.8%)			
White	58 (80.6%)	20 (90.9%)	6 (100%)	5 (100%)	89 (84.8%)	16 (59.3%)	53 (66.3%)	158 (74.5%)			
Unreported	2 (2.8%)	0 (0.0%)	0 (0.0%)	0 (0.0%)	2 (1.9%)	4 (14.8%)	4 (5.0%)	10 (4.7%)			
TBV Harmonized											
Mean (SD)	993 (94)	1,050 (105)	1,180 (34)	1,100 (110)	1,020 (106)	1,230 (116)	1,060 (101)	1,060 (125)	0.021*^b^	<0.001*^b^	<0.001*^d^
Median [Min, Max]	991 [722, 1200]	1,040 [899, 1320]	1,180 [1140, 1230]	1,100 [934, 1240]	1,020 [722, 1320]	1,220 [963, 1450]	1,050 [856, 1340]	1,050 [722, 1450]			

Note. The results are reported on ComBat-harmonized data; values for TBV (mean, SD, median, min, and max) were divided by 1,000. Values with an asterisk (*) represent significant *p* < 0.05. NSSD, Noonan syndrome spectrum disorder; NF1, neurofibromatosis type 1; TBV, total brain volume.

a *p* values calculated using chi-square tests.

b *p* values calculated using Mann–Whitney *U* tests.

c *p* values calculated using Fisher's exact tests.

d *p* values calculated using independent samples *t* tests.

### TBV differences across RASopathies

**Figure 1.**
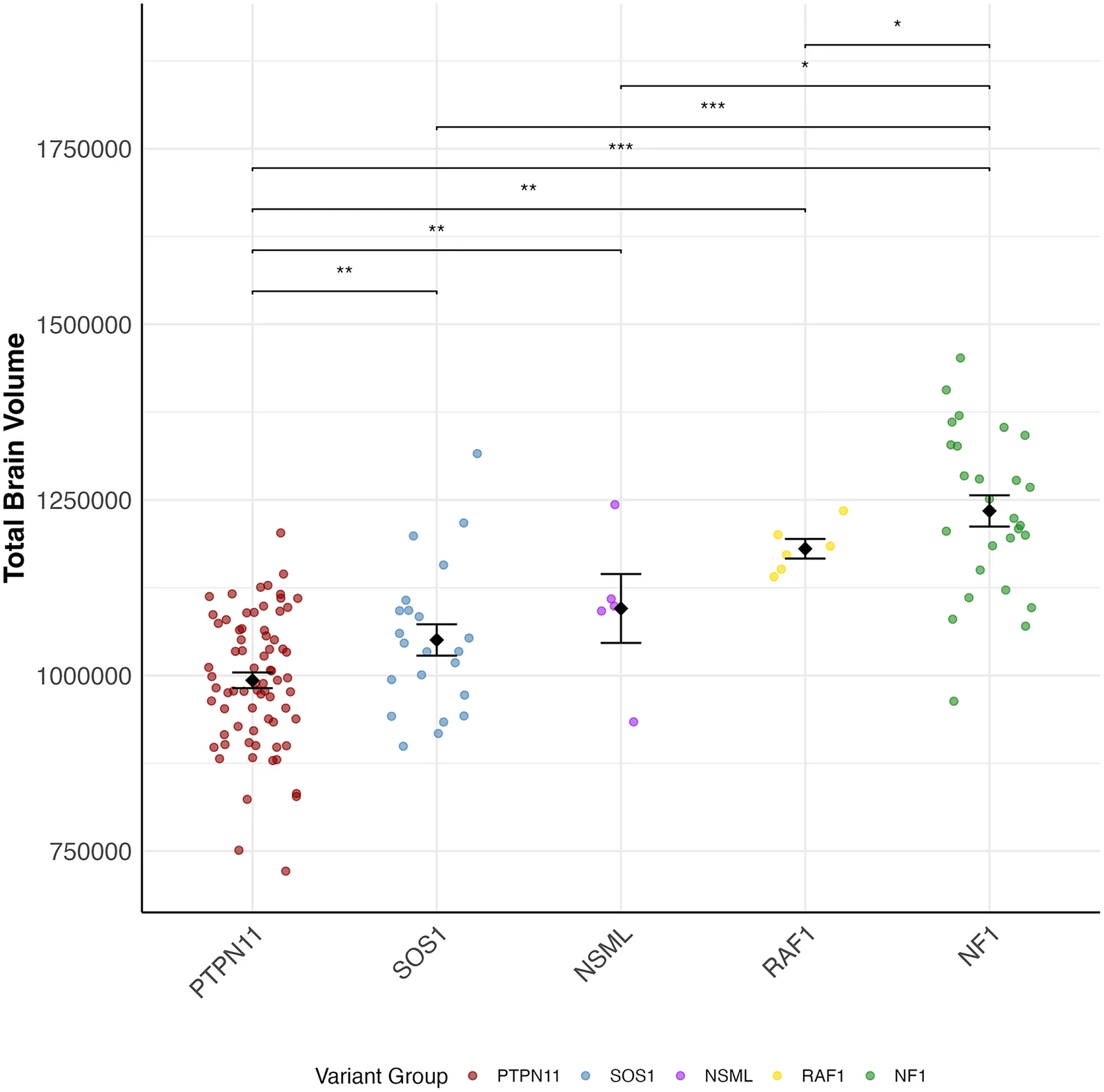
Comparisons between genetic variant groups reveal differences in TBV. *** indicates FDR-corrected *p* < 0.001, **p < 0.010, *p < 0.050. Points and error bars indicate means and standard deviations, respectively. NF1, neurofibromatosis type 1; NSML, Noonan syndrome with multiple lentigines.

First, to test whether TBV varies by genetic variant groups, we compared TBV between variant groups. We observed distinct differences in TBV between RASopathy-associated genetic variant groups (Table 2, Fig. 1). Youth with NF1 consistently exhibited enlarged TBV compared with those with NS and NSML (Table 2, Fig. 1). Similarly, NSML youth showed significantly enlarged TBV compared with *PTPN11* variants (*p*_FDR_ = 0.006). Within the NS-associated genetic variant groups, youth with *RAF1* variants had significantly enlarged TBV compared with those with *PTPN11* (*p*_FDR_ = 0.001), while youth with *SOS1* also exhibited significantly larger TBV compared with *PTPN11* (*p*_FDR_ = 0.006).

**Table 2.** Genetic variant differences across TBV

	NF1	NSML	*PTPN11*	*RAF1*	*SOS1*
NF1	1,234,279 ± 115,629				
NSML	0.018*	1,095,418 ± 109,657			
*PTPN11*	<0.001*	0.006*	993,164 ± 94,522		
*RAF1*	0.026*	0.800	0.001*	1,180,459 ± 34,176	
*SOS1*	<0.001*	0.189	0.006*	0.099	1,050,604 ± 104,772

Note. NF1, neurofibromatosis type 1; NSML, Noonan syndrome with multiple lentigines. Diagonal values show the mean ± standard deviation (SD) for each genetic variant. Off-diagonal values show pairwise group comparison *p* values (FDR-corrected). Values with an asterisk (*) indicate statistically significant differences after FDR correction (*p*_FDR_ < 0.05).

### Allometry of subcortical volumes in TD youth

To then establish a normative framework for brain scaling, we first modeled the allometric relationship between subcortical structures and TBV in TD youth (Table 3). All subcortical regions demonstrated allometric coefficients (*β*) < 1.0, consistent with disproportionately smaller subcortical volumes relative to TBV at higher TBV values. The caudate (*β* = 0.67, *p*_FDR_ = 0.008), putamen (*β* = 0.57, *p*_FDR_ < 0.001), and thalamus (*β* = 0.74, *p*_FDR_ < 0.001) showed significant hypoallometric scaling, meaning that across TD youth, larger brains are associated with subcortical volumes that are proportionally smaller than would be expected under isometric scaling. In contrast, coefficients for the ventral striatum (*β* = 0.80), pallidum (*β* = 0.75), hippocampus (*β* = 0.86), and amygdala (*β* = 0.92) were not significantly different from isometric scaling (*p*s > 0.05).

**Table 3.** Allometry of subcortical volume in TD youth

Structure	Allometric coefficient (β)	95% CI	*p*_FDR_ value
Caudate	0.67	0.43–0.91	0.008*
Putamen	0.57	0.34–0.79	<0.001*
Ventral Striatum	0.80	0.50–1.10	0.192
Thalamus	0.74	0.59–0.88	<0.001*
Pallidum	0.75	0.48–1.01	0.066
Hippocampus	0.86	0.70–1.01	0.076
Amygdala	0.92	0.73–1.11	0.407

Note. Normative allometric coefficients, TD youth-related scaling coefficients followed by 95% confidence intervals, and associated *p* values testing whether the scaling of each subcortical volume with TBV are significantly different from 1. Values with an asterisk (*) represent significant *p* values (*p* < 0.05). Sensitivity analyses controlling for age and imaging pipeline showed a similar pattern (Extended Data Tables 3-1, 3-2).

10.1523/ENEURO.0017-26.2026.t3-1 Table 3-1 **Allometry of subcortical volume in typically developing youth controlling for age.** *Note.* Normative allometric coefficients, typically developing youth-related scaling coefficients followed by 95% confidence intervals, and associated *p*-values for the scaling of each subcortical volume with TBV are significantly different from 1. An asterisk represents significant *p*-values (*p* < 0.05). Download Table 3-1, DOCX file.

10.1523/ENEURO.0017-26.2026.t3-2 Table 3-2 **Allometric estimates of subcortical volume in typically developing youth, controlling for the imaging pipeline.** *Note.* Allometric coefficients (*β*) represent scaling relationships between subcortical volume and TBV, with values significantly different from 1 indicating deviation from isometric scaling. An asterisk represents significant *p*-values (*p* < 0.05). Download Table 3-2, DOCX file.

In sensitivity analyses of the normative allometric models in the TD sample, age and imaging acquisition/processing pipeline were controlled for separately. We observed a similar pattern of hypoallometric scaling in both analyses, indicating that the relationships were robust to age-related effects and variation in scanner type and FreeSurfer version (Extended Data Tables 3-1, 3-2).

### Variant-specific allometric deviations in subcortical volumes

**Figure 2.**
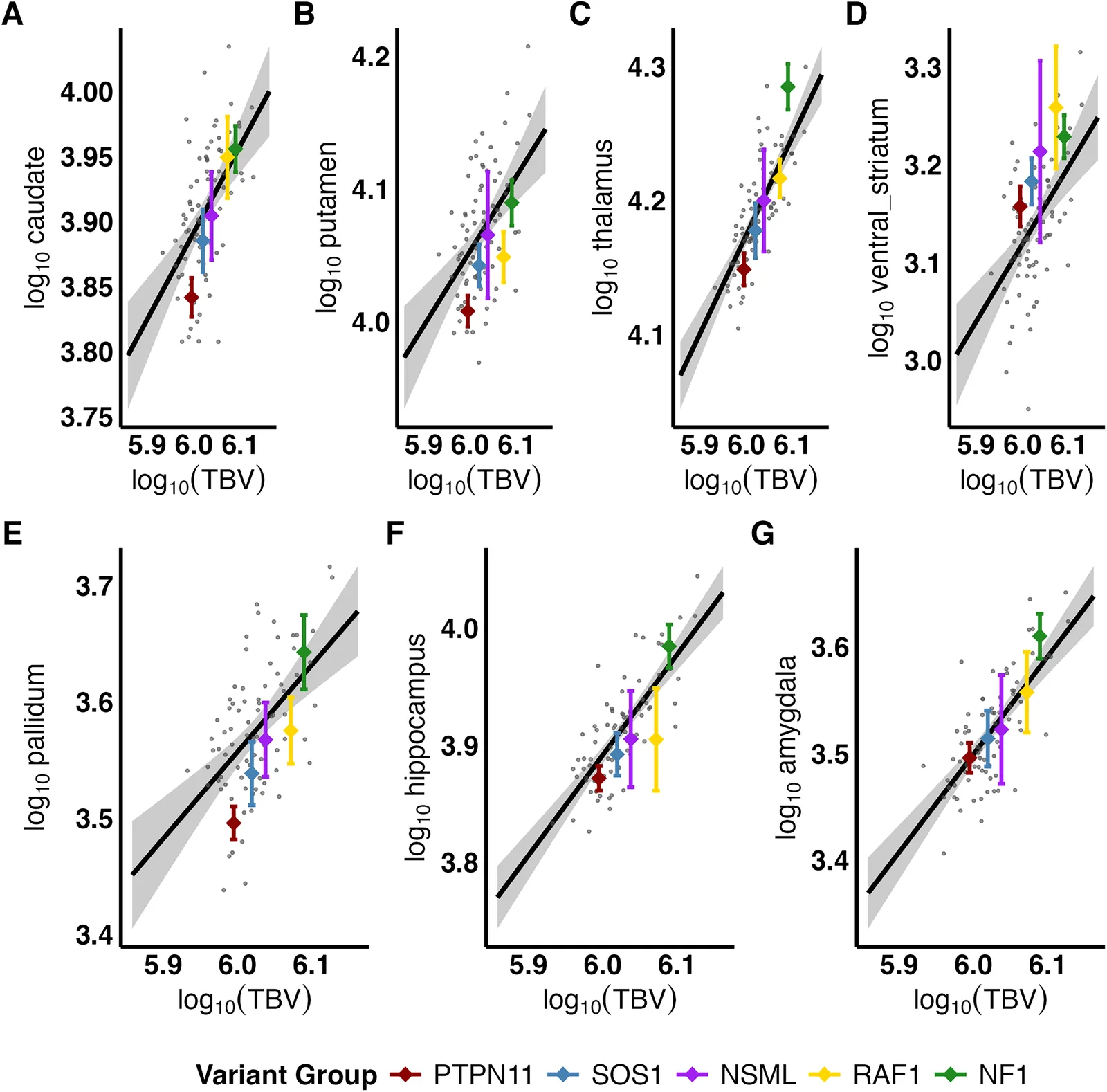
Subcortical volume findings in our core sample in the context of normal brain allometry. Gray points and fit lines (see Table 3 for associated coefficients) show the log–log relationship between TBV and each subcortical volume in the TD sample. Superimposed points show 95% confidence intervals of observed distributions of subcortical volumes (caudate, putamen, thalamus, ventral striatum, pallidum, hippocampus, and amygdala) at mean TBV for each genetic variant group within our core sample (groups color-coded). NF1, neurofibromatosis type 1; NSML, Noonansyndrome with multiple lentigines;TBV, total brain volume*.*

For the first aim, we observed that subcortical volumes in RASopathy genetic variant groups significantly deviated from normative allometric relationships (Fig. 2). Specifically, in youth with *PTPN11*, dorsal striatal structures (caudate and putamen) and pallidum volumes showed negative deviations from normative scaling, indicating that proportional volumes were smaller-than-expected based on TD allometric relationships (Fig. 2). *RAF1* also showed a similar negative deviation in the putamen, with volumes smaller than predicted by TD allometry. In contrast, the ventral striatum in both *PTPN11* and *SOS1* demonstrated positive deviations from normative scaling, indicating that proportional volumes were larger-than-expected relative to TBV. For NF1, the thalamus was significantly larger than predicted relative to TBV, suggesting a positive deviation from normative scaling. However, youth with NSML showed no significant deviations across any subcortical volumes, indicating adherence to the expected growth patterns. Finally, no significant deviations were found in the hippocampus or amygdala in any RASopathy group (Fig. 2*F*,*G*), suggesting those volumes followed normative scaling.

### Allometric sex differences in subcortical volumes

Exploratory sex-stratified analyses within the allometric framework revealed differences in normative allometric scaling relationships within the TD sample (Table 4). Females exhibited hypoallometric scaling in several subcortical regions, including the putamen, ventral striatum, thalamus, pallidum, and hippocampus, indicating disproportionately smaller subcortical volumes relative to TBV at higher TBV values. In contrast, males generally exhibited isometric scaling across subcortical regions.

**Table 4.** Allometry of subcortical volumes in TD females and males

Structure	Allometric coefficient (β)	95% CI	*p*-value
Females			
Caudate	0.73	0.40–1.05	0.110
Putamen	0.47	0.20–0.75	<0.001*
Ventral striatum	0.54	0.13–0.95	0.035*
Thalamus	0.69	0.52–0.86	0.001*
Pallidum	0.62	0.27–0.98	0.042*
Hippocampus	0.75	0.53–0.98	0.039*
Amygdala	0.85	0.57–1.13	0.307
Males			
Caudate	0.76	0.37–1.15	0.246
Putamen	0.67	0.25–1.09	0.132
Ventral striatum	1.06	0.57–1.56	0.805
Thalamus	0.82	0.54–1.10	0.205
Pallidum	0.94	0.47–1.40	0.788
Hippocampus	1.03	0.81–1.26	0.768
Amygdala	1.01	0.70–1.32	0.938

Note. TBV, total brain volume*.* Normative allometric coefficients, TD individual-related scaling coefficients, and associated *p* values testing whether the scaling of each subcortical nucleus volume with TBV are significantly different from 1. Values with an asterisk (*) represent significant *p* values after FDR correction (*p*_FDR _< 0.05). Sex-stratified deviations from the TD-derived normative models are provided in Extended Data Table 4-1.

10.1523/ENEURO.0017-26.2026.t4-1 Table 4-1 **Sex-stratified standardized deviations from TD-derived normative allometric scaling relationships** *Note.* Values represent standardized deviations from the sex-specific TD-derived normative allometric scaling models. Deviations were calculated as the difference between observed and TD-predicted subcortical volumes, divided by the residual standard deviation of the corresponding sex-specific TD model. Positive values indicate larger-than-expected proportional volumes relative to TBV, whereas negative values indicate smaller-than-expected proportional volumes relative to TBV. Values are presented as Female, Male. Download Table 4-1, DOCX file.

**Figure 3.**
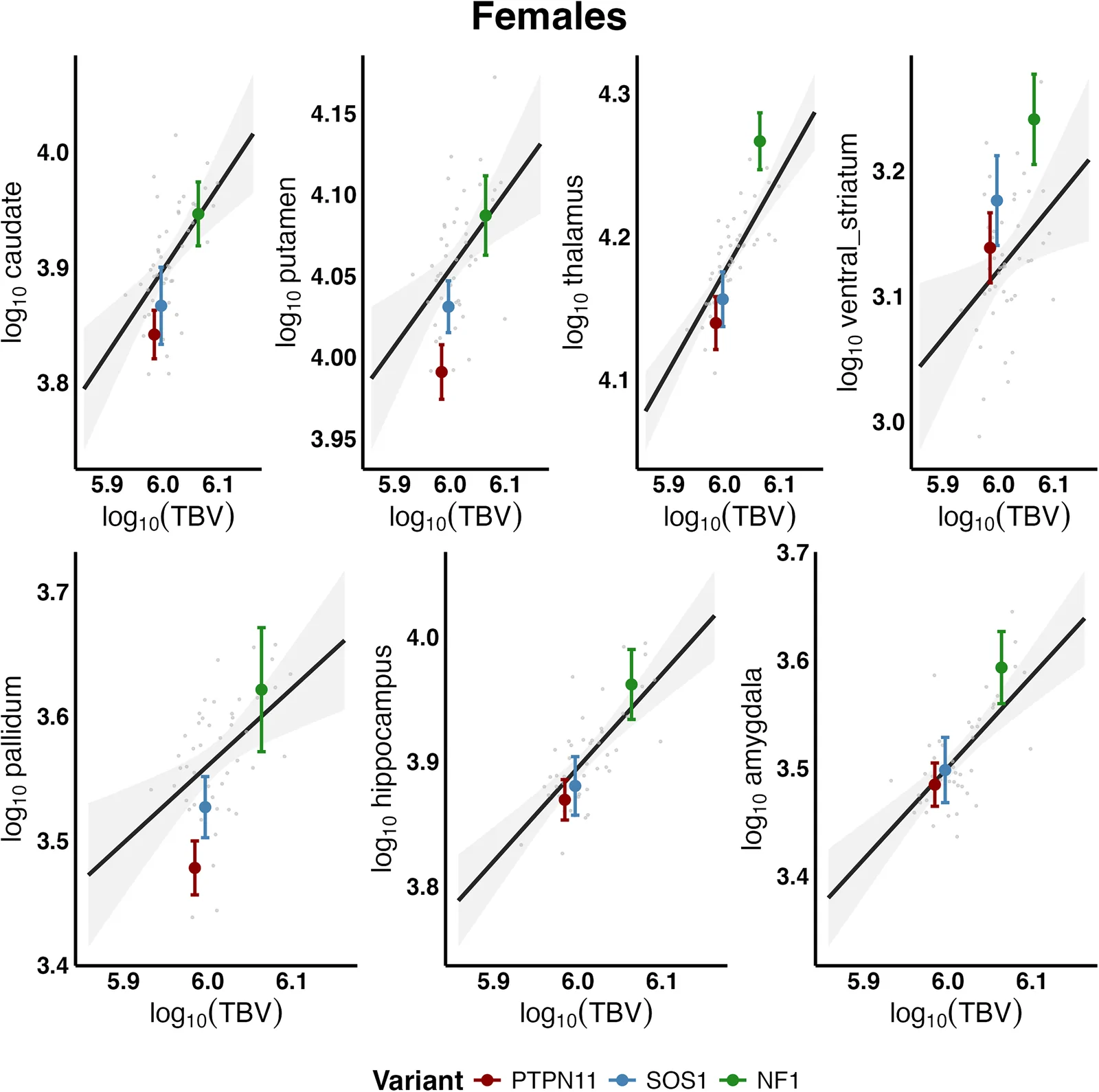
Subcortical volume findings in our core female sample in the context of normal brain allometry. Black fit lines (see Table 4 for associated coefficients) show the log–log relationship between TBV and each subcortical volume in the TD female sample. The shaded light gray regions represent the 95% confidence intervals (CIs) for TD females, and the light gray points represent the individual log–log observations for each TD female. Superimposed points show the 95% confidence intervals of observed distributions of subcortical volumes at the mean TBV for each genetic variant group within our core sample of females (groups color-coded). NF1, neurofibromatosis type 1; TBV, total brain volume*.*

**Figure 4.**
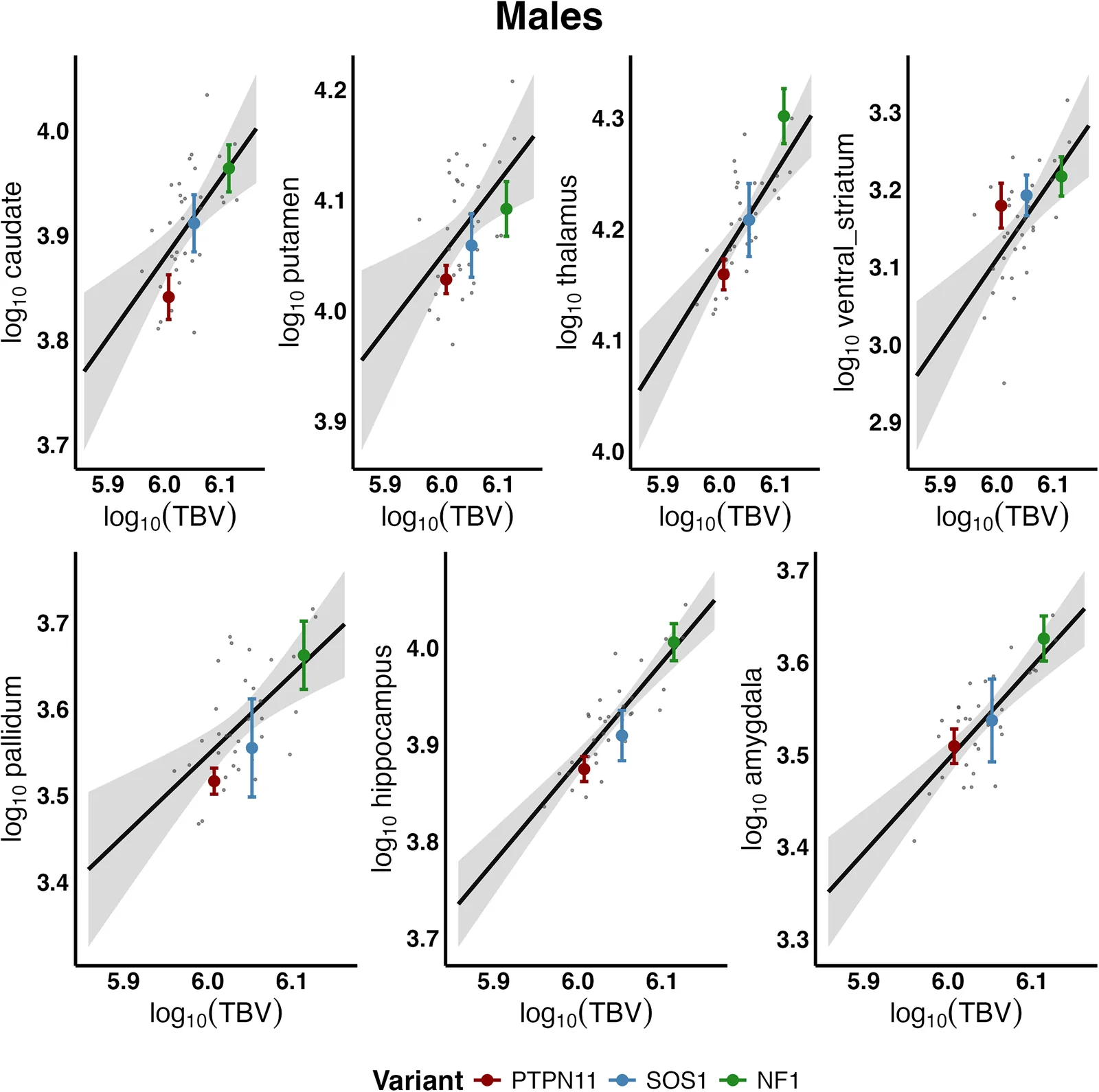
Subcortical volume findings in our core male sample in the context of normal brain allometry. Black fit lines (see Table 4 for associated coefficients) show the log–log relationship between TBV and each subcortical volume in the TD male sample. The shaded light gray regions represent the 95% confidence intervals (CIs) for TD males, and the light gray points represent the individual log–log observations for each TD male. Superimposed points show the 95% confidence intervals of observed distributions of subcortical volumes at the mean TBV for each genetic variant group within our core sample of males (groups color-coded). NF1, neurofibromatosis type 1; TBV, total brain volume.

Relative to these sex-specific TD normative models, region-specific deviations were observed across genetic variants (Figs. 3, 4, Extended Data Table 4-1). While some regional differences in the magnitude of deviation from normative scaling were observed between sexes, these patterns were not consistent across subcortical regions or genetic groups.

As shown in Figures 3 and 4, in *PTPN11*, both females and males showed negative deviations in the caudate (females, *z* = −1.08; males, *z* = −0.95), indicating smaller-than-expected proportional volumes relative to TBV. Females additionally showed larger negative deviations in the putamen (*z* = −1.64) and pallidum (*z* = −1.62) compared with smaller deviations in males (putamen, *z* = −0.54; pallidum, *z* = −0.69). In contrast, males showed a positive deviation in the ventral striatum (*z* = 1.05), with a smaller deviation in females (*z* = 0.51). In NF1 and *SOS1*, positive deviations were more pronounced in females. In NF1, females showed positive deviations in the ventral striatum (*z* = 1.66) and thalamus (*z* = 2.24), whereas males showed smaller deviations (ventral striatum, *z* = −0.25; thalamus, *z* = 1.19), which were not statistically significant. In *SOS1*, females showed a positive deviation in the ventral striatum (*z* = 1.10), with a smaller deviation in males (*z* = 0.46), which was not statistically significant. No other subcortical regions showed statistically significant deviations from TD-derived normative scaling. However, given the modest subgroup sample sizes, these analyses were not powered to determine whether the observed allometric patterns are sex-specific and should therefore be considered preliminary.

### Comparison of different methods of controlling for brain size across subcortical volumes

In line with our observation that some subcortical volumes scale nonlinearly relative to TBV (Table 3), we found that linear TBV correction methods, such as normalization and covariation, might overestimate regional effects because of their linear scaling assumptions (Table 5).

**Table 5.** Comparing each genetic variant group with TD younth across different methods of controlling for brain size in the analysis of subcortical volume

	TD versus					
	NF1	*PTPN11*	*SOS1*	*RAF1*	NSML
Caudate						
Normalization	**—**	**↓**	**—**	**—**	**—**
Covariation	**—**	**↓**	**—**	**—**	**—**
Allometry	**—**	**↓**	**—**	**—**	**—**
Putamen						
Normalization	**↓**	**↓**	**—**	**↓**	**—**
Covariation	**—**	**↓**	**↓**	**↓**	**—**
Allometry	**—**	**↓**	**—**	**↓**	**—**
Ventral Striatum						
Normalization	**↑**	**↑**	**↑**	**↑**	**↑**
Covariation	**↑**	**↑**	**↑**	**↑**	**↑**
Allometry	**—**	**↑**	**↑**	**—**	**—**
Thalamus						
Normalization	**↑**	**↓**	**—**	**—**	**—**
Covariation	**↑**	**↓**	**—**	**—**	**—**
Allometry	**↑**	**—**	**—**	**—**	**—**
Pallidum						
Normalization	**—**	**↓**	**↓**	**↓**	**—**
Covariation	**—**	**↓**	**↓**	**—**	**—**
Allometry	**—**	**↓**	**—**	**—**	**—**
Hippocampus						
Normalization	**—**	**—**	**—**	**↓**	**—**
Covariation	**↑**	**↓**	**↓**	**↓**	**—**
Allometry	**—**	**—**	**—**	**—**	**—**
Amygdala						
Normalization	**↑**	**—**	**—**	**—**	**—**
Covariation	**↑**	**—**	**—**	**—**	**—**
Allometry	**—**	**—**	**—**	**—**	**—**

Note. NF1, neurofibromatosis type 1; NSML, Noonan syndrome with multiple lentigines; TD, typically developing youth. Subcortical volumes were compared between each genetic variant group and TD youth using three methods to control for total brain size variation: normalization by TBV, covariation for TBV, and use of the allometric framework. Arrows indicate the presence and direction of statistically significant group differences in the volume of each structure relative to TD youth, with *p_FDR _*< 0.05.

In NF1, we found consistent significant differences across all three correction methods in the thalamus. The allometric approach yielded no additional differences, whereas linear correction methods identified differences in the putamen, ventral striatum, hippocampus, and amygdala, indicating that estimates in these regions may be sensitive to linear TBV correction. In contrast, thalamic estimations were robust to all three correction methods.

Among NS-associated variants, *PTPN11* demonstrated significant differences across all three correction methods in the dorsal striatum (caudate, putamen), ventral striatum, and pallidum, indicating region-specific genetic effects that are robust to linear and allometric correction methods. In contrast, *SOS1* and *RAF1* showed inconsistent results across methods. Specifically, *SOS1* showed significant differences across all three correction methods in the ventral striatum, whereas the results for the putamen and pallidum were not consistent across methods. Similarly, for *RAF1*, results converged for the putamen; however, outcomes for the ventral striatum and pallidum diverged depending on the correction method.

Moreover, in NSML, the ventral striatum appeared significantly larger than TD volumes in both normalization and covariation analyses but showed no deviation in the allometric approach, suggesting sensitivity of this finding to scaling assumptions.

Lastly, when comparing subcortical volumes across RASopathy groups, rather than relative to controls, normalization and covariation linear correction methods generally showed convergent results (Table 6), with the majority of significant group differences identified consistently across methods. The few divergent results that emerged suggest that certain subcortical findings are sensitive to the assumptions underlying the TBV correction method, particularly for the caudate, putamen, hippocampus, and thalamus.

**Table 6.** Comparison of genetic variant groups with one another across different methods of controlling for brain size in the analysis of subcortical volume

	*PTPN11* versus *SOS1*	*PTPN11* versus NF1	*PTPN11* versus NSML	*PTPN11* versus *RAF1*	*SOS1* versus NF1	*SOS1* versus NSML	*SOS1* versus *RAF1*	NF1 versus NSML	NF1 versus *RAF1*	NSML versus *RAF1*
Volumes	*p_FDR_* values									
Caudate										
Normalization	0.216	0.178	0.640	0.265	0.968	0.968	0.770	0.968	0.758	0.758
Covariation	0.121	**0.034↓**	0.369	0.121	0.185	0.819	0.273	0.424	0.819	0.427
Putamen										
Normalization	0.369	0.240	0.530	0.072	0.112	0.902	**0.044↓**	0.240	0.240	**0.008↑**
Covariation	0.070	0.105	0.155	0.484	0.213	0.423	0.664	0.846	0.115	**0.021↑**
Ventral Striatum										
Normalization	0.943	0.256	0.820	0.598	0.328	0.813	0.588	0.368	0.219	0.871
Covariation	0.915	0.915	0.784	0.531	0.329	0.546	0.127	0.908	0.423	0.915
Thalamus										
Normalization	0.681	**<0.001↓**	0.681	0.613	**0.002↓**	0.851	0.453	0.101	**0.006↓**	0.303
Covariation	0.493	**<0.001↓**	0.505	0.904	**0.001↓**	0.702	0.960	**0.047↓**	**0.006↓**	0.628
Pallidum										
Normalization	0.223	**<0.001↓**	0.359	0.987	0.164	0.828	0.529	0.529	0.223	0.216
Covariation	0.143	**0.001↓**	0.173	0.417	0.135	0.675	0.822	0.417	0.215	0.422
Hippocampus										
Normalization	0.742	0.055	0.621	**0.025↓**	0.097	0.742	0.097	0.156	**0.004↓**	0.213
Covariation	0.623	**<0.001↓**	0.852	0.344	**0.005↓**	0.895	0.623	0.101	**0.005↓**	0.344
Amygdala										
Normalization	0.710	0.117	0.591	0.591	0.114	0.710	0.710	0.120	0.114	0.978
Covariation	0.899	0.112	0.822	0.861	0.138	0.861	0.945	0.232	0.189	0.861

Note. Subcortical volumes were compared between each genetic variant group using the two linear correction methods to control for total brain size variation: normalizing by TBV and covariation by TBV. Bolded *p*_FDR_ values alongside arrows indicate the presence and direction of statistically significant group differences in the volume of each structure at *p*_FDR_ < 0.05. The direction of each arrow reflects the outcome of the comparison. Specifically, an upward arrow (↑) indicates that the group listed first in the comparison has significantly larger volumes than the group listed second, while a downward arrow (↓) indicates that the first group has significantly smaller volumes than the second group.

## Discussion

In the present study, we used an allometric framework to determine whether (1) subcortical volumes show allometric deviations from normative scaling, which provides insights into the biological scaling of subcortical structures in RASopathies, and (2) the methods used to account for TBV influence the detection of subcortical volume alterations in RASopathies. RASopathies provide a useful model for addressing this question because they are characterized by substantial alterations in TBV alongside well-established subcortical volume alterations (Johnson et al., 2019; Rai et al., 2023; McGhee et al., 2024; Serur et al., 2025a). First, in TD youth, the caudate, putamen, and thalamus showed hypoallometric scaling, indicating that proportional subcortical volumes were smaller with increasing TBV. Relative to this normative scaling pattern established in TD youth, RASopathy variant groups showed distinct deviations: *PTPN11* was associated with negative deviations in the caudate, putamen, and pallidum, indicating subcortical volumes that were smaller-than-expected based on normative scaling relationships, and a positive deviation in the thalamus, whereas NF1 showed positive deviations in the thalamus, indicating larger-than-expected proportional volumes relative to TBV. Comparisons across correction methods indicated that linear approaches may overestimate some regional differences. Still, thalamic effects in NF1 and striatal effects in *PTPN11* were consistent across methods, suggesting robust, region-specific genetic effects. Together, these findings refine the interpretation of neuroanatomical differences in RASopathies and highlight the importance of accounting for nonlinear brain–size scaling in neuroimaging studies.

### Importance of allometric scaling for RASopathies

While prior neuroimaging studies of RASopathies have reported subcortical volume differences relative to TD individuals, most have relied on linear correction methods, such as normalization or covariation with TBV (Violante et al., 2013; Barkovich et al., 2018; Johnson et al., 2019; Rai et al., 2023; McGhee et al., 2024; Siqueiros-Sanchez et al., 2024; Serur et al., 2025a). These methods assume linear scaling between regional structures and TBV, which can overestimate region-specific genetic effects by obscuring them with scaling-related differences, especially in populations where TBV is known to be altered (Violante et al., 2013; Johnson et al., 2019; Wang et al., 2022). In contrast, our findings reveal that some subcortical structures scale nonlinearly with TBV, aligning with previous research on normative brain development (Reardon et al., 2016). We established normative allometric relationships in TD individuals and found that the particularly the caudate, putamen, and thalamus, exhibit hypoallometric scaling, indicating that proportional subcortical volumes become smaller with increasing TBV.

Distinct deviations emerged across RASopathy genetic variant groups after allometric adjustments, highlighting variant-specific patterns of subcortical scaling. The most pronounced and widespread deviations from normative scaling were observed in youth with *PTPN11*, particularly in the dorsal striatum (caudate, putamen), pallidum, and ventral striatum. These findings are consistent with previous research findings that subcortical reductions in *PTPN11*, which disrupt SHP2 function and neural progenitor cell fate, may alter neuron-to-glia ratios, contributing to smaller volumes (Gauthier et al., 2007; Johnson et al., 2019; Payne et al., 2025). In contrast, deviations were more regionally specific in other variants, localized to the thalamus in NF1, the ventral striatum in *SOS1*, and the putamen in *RAF1*, whereas youth with NSML showed no significant deviations, indicating relative normative scaling of subcortical volumes. Although NF1, NS, and NSML all involve dysregulation of RAS/MAPK signaling, they arise from distinct genetic variants that differ in their molecular mechanisms and may also differentially affect related pathways (Rauen, 2013; Tidyman and Rauen, 2016b), including PI3K/AKT/mTOR signaling. While the present study does not assess molecular mechanisms directly, the observed neuroanatomical differences are consistent with this underlying genetic and biological heterogeneity across RAS/MAPK-related disorders. Future work leveraging larger variant groups will be critical to validate these patterns and further clarify their functional and clinical relevance.

Convergence between linear correction methods and allometric approaches was observed, though it varied across subcortical volume regions and genetic variant groups. When findings were consistent across methods, this suggests that the observed group differences reflect robust region-specific effects that are not attributable to the choice of scaling method. For example, several alterations remained evident across correction approaches, including caudate and pallidal alterations across multiple RASopathy-associated variants and thalamic differences in NF1, supporting the interpretation that these represent robust genetic effects. In contrast, discrepancies across methods indicate that apparent group differences are sensitive to assumptions about how regional volumes scale with TBV. These findings reinforce the growing recognition that nonlinear scaling relationships must be explicitly modeled to accurately interpret neuroanatomical differences in neurogenetic syndromes and psychiatric conditions (Reardon et al., 2016; Nadig et al., 2018; Bethlehem et al., 2022; Janssen et al., 2022). Given that RASopathies are characterized by altered TBV, these findings suggest that some previously reported subcortical alterations likely reflect genetic effects, whereas others may be influenced by TBV scaling assumptions. As personalized interventions for youth with RASopathies continue to be refined, incorporating allometric models may help identify biologically meaningful deviations from normative brain growth trajectories. In turn, such allometrically informed findings may provide a useful approach for monitoring neurodevelopmental trajectories.

### Preliminary sex-specific distinction in normative scaling

Our study is the first, to our knowledge, to systematically investigate sex-specific differences in subcortical allometric scaling and their relevance to RASopathies. These analyses were exploratory, given the limited statistical power to detect subtle differences in scaling relationships. In TD youth, we identified sex differences in scaling patterns: females showed more pronounced hypoallometric scaling across subcortical regions, whereas males exhibited more proportional, normative scaling relative to TBV. These findings align with previous evidence that sex may influence the relationship between TBV and subcortical volume (Reardon et al., 2016). We then examined whether deviations from normative allometric scaling differed between males and females in RASopathy-associated variant groups. While some variation in effect magnitude was observed across sexes, these patterns were not consistent across regions or genetic variant groups.

Effects should be interpreted cautiously, given limited statistical power, particularly within variant-specific analyses. Although some allometric deviations appeared more pronounced in females, these exploratory analyses were conducted in relatively small variant-specific groups and were not designed to formally test sex-specific effects. Consequently, it remains unclear whether the observed differences reflect biological heterogeneity, sampling variability, or reduced precision associated with subgroup analyses. Larger studies will be required to determine whether deviations from normative scaling differ by sex in RASopathies and to establish the extent to which sex contributes to the neuroanatomical patterns observed in the full sample.

### Limitations and future directions

There are limitations to our present study. Most importantly, for our rarest variants, our sample sizes were small and unbalanced. Given the rarity of these groups, it is difficult to recruit large samples. Global efforts across lifespan studies may also aid in increasing sample sizes of these groups. Future studies should aim to include larger numbers of participants with these rarer genetic variants causing NS or NSML to increase statistical power and enable more reliable subgroup analyses. 

Additionally, our TD participants were recruited locally, which may limit the generalizability of the allometric scaling reference group. However, recruiting TD youth from the same site and cohort as the RASopathy groups helped minimize measurement-related confounds. While we found no differences in demographics between the NF1 and TD groups, we did observe demographic differences between the NSSD and TD groups, with the NSSD group being predominantly of European ancestry. Future studies should aim to include a more demographically and geographically diverse TD sample to enhance the generalizability of these findings.

It is important to note that this study is cross-sectional, which limits the generalizability of our findings across different stages of development. Although the age of participants was not significantly different across groups, future research should apply this allometric framework in a longitudinal design to explore time-specific alterations in subcortical volumes. Moreover, the rarity of RASopathies poses a challenge in recruiting adequate cohorts for longitudinal analyses, highlighting the need for ongoing research in this area.

Finally, while the precise mechanisms remain unclear, the variant-specific influences we observed likely reflect differences in underlying molecular pathways. For example, consistent subcortical reductions in *PTPN11*, whose variants disrupt SHP2 function and neural progenitor cell fate, may alter neuron-to-glia ratios, contributing to smaller volumes (Gauthier et al., 2007; Johnson et al., 2019; Payne et al., 2025). Future studies, such as gene expression studies in human neural tissue, should investigate whether SHP2-related alterations contribute to regional variations in RASopathy scaling differences.

### Conclusions

This study underscores the importance of applying allometric methods in neuroanatomical research, particularly in conditions characterized by atypical brain size, such as RASopathies. By accounting for nonlinear scaling, allometric approaches allow us to accurately estimate region-specific neuroanatomical differences that reflect genetic influences that linear correction methods may overestimate. As we work to enhance personalized interventions for youth with RASopathies, it is essential to consider their unique brain development, as these factors may in the future serve as important outcome measures. Our findings deepen the understanding of neuroanatomical differences in RASopathies by revealing subcortical regions that significantly deviate from normative scaling patterns. Ultimately, these findings emphasize the need to incorporate normative allometry in the evaluation of neuroanatomy across genetic and psychiatric conditions known to influence TBV.

## Synthesis

Reviewing Editor: Gergely Silasi, University of Ottawa Decisions are customarily a result of the Reviewing Editor and the peer reviewers coming together and discussing their recommendations until a consensus is reached. When revisions are invited, a fact-based synthesis statement explaining their decision and outlining what is needed to prepare a revision will be listed below. The following reviewer(s) agreed to reveal their identity: NONE. Note: If this manuscript was transferred from JNeurosci and a decision was made to accept the manuscript without peer review, a synthesis may not be available.

An expert reviewer has provided several suggestions, most notably on the statistical analyses and elimination of potential confounds that may impact interpretation of the results. Please provide point-by-point responses to these queries and we will re-review the revised manuscript.

Overall Review:

This paper investigates non-linear relationships between subcortical volume and total brain volume between a clinical sample of RASopathies and typically developing children and youth. It is definitely important that rare genetic conditions are researched and understood even if sample sizes are small. The strengths of this study are: an appropriately sized sample of rare genetic conditions, a meaningful control group serving as a preliminary reference point, and novelty in addressing nonlinear neurobiological relationships. The weaknesses of the study is the lack of conceptual integration of all these components making it difficult to understand what this study actually sought out to do, why it is important, and the utility of the results. Below, I provide comments on specific points of weakness that I recommend improving to increase the utility and impact of this paper.

Introduction:

RASopathies are described as rare genetic conditions in the introduction, but there is no explanation of the clinical manifestations of these conditions. Without this information, it is difficult to understand why subcortical structures were the focus of this study. I recognize that the authors mentioned prior work has found alterations in subcortical structures, however, if the purpose of the paper is to understand case-control differences, it's important for a reader to have more information about the clinical group of interest.

Related to the first point, the way the paper is structured makes it difficult to understand whether the main purpose of the paper is to compare traditional linear scaling and nonlinear scaling OR whether it is to understand the nonlinear nature of subcortical volume alterations in RASopathies. I was hoping for a bit more clarity as I read the paper, but did not find it. I recommend that the authors clarify the conceptual links between these points in the introduction so the reader has a framework for the purpose of the paper and can interpret the methods and results accordingly.

I liked this sentence in the introduction: Together, these studies highlight the importance of allometric approaches for conditions with atypical brain size, capturing nonlinear scaling and region-specific effects that traditional methods may overestimate. I think that the ideas here need to be better substantiated throughout the introduction so this sentence is a logical conclusion based on the conceptual ideas provided earlier.

Methods:

Can the authors clarify that participants were recruited all over the US and Canada but scanned in one site? Were they treated at this site or did they come all the way to participate in this study?

What does "molecularly confirmed diagnosis" mean? Is this different from genetically diagnosed patients? For readers who are not familiar with these conditions (which I assume will be many), these details are helpful in providing context to the decisions of the study.

I recognize constraints of scanner upgrades and harmonizing scans collected from 2 different scanners, but it is unclear why two different versions of FreeSurfer were used. Given the inevitable variation in ROIs when using different pipelines, each added difference to the processing and analytical pipeline increases the risk of more unmeasured variation. I recognize that it's a lot to ask to re-process all the scans and redo the analyses, but at the very least, it is critical for authors to explore any potential variations in subcortical volumes and TBV between the two subsets using different FreeSurfer versions to see whether that is contributing unintended variation.

The motivation for the sex-specific analysis is not clear. Are there sex differences in neurobiological or clinical manifestations in RASopathies? I think this should be mentioned in the introduction.

For the log-log regression equation, is there a reason that no covariates are included? Presumably, age would influence the nonlinear growth relationship between subcortical structures and TBV.

The methods section reads as a methodological comparison study making it unclear whether there is specific utility in using this clinical cohort. This is the challenge when the introduction does not clarify how the between the different components of the study integrate.

Results:

In the results and discussion, the authors describe allometric findings as "subcortical volumes increase at a slower rate than TBV", however, given that this is a cross-sectional study design, I think it's more accurate that the results revealed that subcortical volumes were disproportionately smaller than TBV.

The sex-specific results introduce an additional complexity when interpreting the results. If the hypoallometric scaling is found in females but not males, that suggests to me the trends found in the full sample are driven by the females. Thus, allometric scaling may be a sex-specific finding, as opposed to being driven by genetic mutations underlying these diagnoses.

Discussion:

Overall, the discussion has the same weakness as the introduction in that the lack of clarity of the framing makes it difficult to contextualize the results. Take the first sentence: In the present study, we examined subcortical volume differences in youth with RASopathies (NF1, NS, and NSML) using normative allometric scaling to more accurately characterize how subcortical structures vary in relation to total brain volume and to address limitations of traditional linear correction methods. This sentence does not make it clear whether the goal is to study case-control subcortical structures or study the variation in scaling of subcortical structures. If the second, it's unclear why this was examined in a rare genetic population versus TD.

An important result that was not well-discussed is the finding that some subcortical structures show similar results with normative and allometric scaling. The authors should provide some insight and intuition as to why they think that is the case, and when allometric scaling provides important insight.

In the discussion, the authors state: Together, these findings highlight variant-specific profiles of subcortical organization within RASopathies and suggest that distinct molecular alterations within the RAS/MAPK pathway may differentially influence neuroanatomical development. Given that there was no assessment of molecular alterations, this statement is not substantiated. I think it's okay to speculate a relationship, but the results, as it stands, do not suggest this.

I also found a few other statements in the discussion that are not substantiated based on the results. I recommend changing the language when describing the importance of the results so it is focused on what was found.

Similar to my last point in the results feedback, the sex-specific findings present a tension with the claim the authors are making that allometric relationships may be important for genetic conditions. It is important that the authors specifically address this tension to help the reader interpret the results.
