# Supplementary material for: An Allometric Approach to Fine-Mapping Subcortical Volume Alterations in RASopathies
Source: eNeuro. 2026 Sep 18;13(9):ENEURO.0017-26.2026. doi: 10.1523/ENEURO.0017-26.2026 (PMC13592736; doi:10.1523/ENEURO.0017-26.2026)
Supplement: Supplementary file 1 [file eneuro-13-ENEURO.0017-26.2026-s003.docx]

**Table 3-1: Allometry of subcortical volume in typically developing youth controlling for age**

| **Structure** | **Allometric Coefficient (β)** | **95% CI** | ***p*_FDR_-value** |
| --- | --- | --- | --- |
| Caudate | 0.68 | 0.43-0.93 | 0.014* |
| Putamen | 0.53 | 0.30-0.77 | <0.001* |
| Ventral Striatum | 0.92 | 0.63-1.22 | 0.621 |
| Thalamus | 0.76 | 0.61-0.91 | 0.003* |
| Pallidum | 0.68 | 0.40-0.95 | 0.025* |
| Hippocampus | 0.77 | 0.62-0.92 | 0.003* |
| Amygdala | 0.86 | 0.66-1.05 | 0.157 |

*Note.* Normative allometric coefficients, typically developing youth-related scaling coefficients followed by 95% confidence intervals, and associated *p*-values for the scaling of each subcortical volume with TBV are significantly different from 1. An asterisk represents significant *p*-values (*p*<0.05).
