# Supplementary material for: An Allometric Approach to Fine-Mapping Subcortical Volume Alterations in RASopathies
Source: eNeuro. 2026 Sep 18;13(9):ENEURO.0017-26.2026. doi: 10.1523/ENEURO.0017-26.2026 (PMC13592736; doi:10.1523/ENEURO.0017-26.2026)
Supplement: Supplementary file 2 [file eneuro-13-ENEURO.0017-26.2026-s004.docx]

**Table 3-2: Allometric estimates of subcortical volume in typically developing youth, controlling for the imaging pipeline**

| **Structure** | **Allometric Coefficient (*β*)** | **95% CI** | ***p_FDR_*-value** |
| --- | --- | --- | --- |
| Caudate | 0.66 | 0.43-0.89 | 0.005* |
| Putamen | 0.55 | 0.34-0.77 | <0.001* |
| Ventral Striatum | 0.80 | 0.50-1.10 | 0.196 |
| Thalamus | 0.74 | 0.59-0.88 | 0.001* |
| Pallidum | 0.74 | 0.47-1.01 | 0.058 |
| Hippocampus | 0.86 | 0.70-1.01 | 0.075 |
| Amygdala | 0.92 | 0.72-1.11 | 0.395 |

*Note.* Allometric coefficients (*β*) represent scaling relationships between subcortical volume and TBV, with values significantly different from 1 indicating deviation from isometric scaling. An asterisk represents significant *p*-values (*p*<0.05).
