# Supplementary material for: An Allometric Approach to Fine-Mapping Subcortical Volume Alterations in RASopathies
Source: eNeuro. 2026 Sep 18;13(9):ENEURO.0017-26.2026. doi: 10.1523/ENEURO.0017-26.2026 (PMC13592736; doi:10.1523/ENEURO.0017-26.2026)
Supplement: Supplementary file 3 [file eneuro-13-ENEURO.0017-26.2026-s005.docx]

**Table 4-1: Sex-stratified standardized deviations from TD-derived normative allometric scaling relationships**

| **Structure** | **NF1** | ***PTPN11*** | ***SOS1*** |
| --- | --- | --- | --- |
|  | ***Standardized Deviations*** | | |
| Caudate | 0.05, -0.02 | -1.08, -0.95 | -0.70, -0.15 |
| Putamen | 0.08, -0.68 | -1.64, -0.54 | -0.63, -0.52 |
| Ventral Striatum | 1.66, -0.25 | 0.51, 1.05 | 1.10, 0.46 |
| Thalamus | 2.24, 1.19 | -1.19, -0.53 | -0.80, -0.14 |
| Pallidum | 0.48, 0.17 | -1.62, -0.69 | -0.70, -0.75 |
| Hippocampus | 0.66, 0.25 | -0.49, -0.55 | -0.43, -0.99 |
| Amygdala | 1.09, 0.46 | -0.08, 0.20 | 0.01, -0.29 |

*Note.* Values represent standardized deviations from the sex-specific TD-derived normative allometric scaling models. Deviations were calculated as the difference between observed and TD-predicted subcortical volumes, divided by the residual standard deviation of the corresponding sex-specific TD model. Positive values indicate larger-than-expected proportional volumes relative to TBV, whereas negative values indicate smaller-than-expected proportional volumes relative to TBV. Values are presented as Female, Male.
